# Studying factual versus social cues as triggers of change in food behaviour

**DOI:** 10.1017/jns.2024.82

**Published:** 2024-12-03

**Authors:** Carolin V. Zorell, Ansung Kim, Nicklas Neuman

**Affiliations:** 1 School of Humanities, Education and Social Sciences, Örebro University, Örebro, Sweden; 2 School of Hospitality, Culinary Arts and Meal Science, Örebro University, Grythyttan, Sweden; 3 Department of Food Studies, Nutrition and Dietetics, Uppsala University, Uppsala, Sweden

**Keywords:** Behaviour change, Dietary behaviour, Plant-based, Randomised controlled trial, Social influence, RCT, Randomised controlled trial

## Abstract

Numerous public initiatives aim to influence individual food choices by informing about what is considered ‘healthy’, ‘climate-friendly’, and generally ‘sustainable’ food. However, research suggests that rather than public authorities, social influence is more likely to affect people’s behaviour. Using a randomised controlled trial, this study investigated if and how the two kinds of influences (factual versus social) could affect the real-life, self-reported intake of plant- and animal-based foods. In a four-month randomised controlled trial, a self-selected sample of adults living in Sweden (N = 237) tracked their daily food consumption several times per week using a tailored mobile phone app. Participants were randomised into one of three groups: two treatment groups receiving factual or social information about plant- and animal-based food consumption, or a control group receiving no information. Pre- and post-questionnaires provided additional background information about the participants. Participants’ food habits varied from week to week, and an explorative analysis pointed to a slight decrease in the consumption of animal-based food in the group that received social information. However, the longer-term patterns remained relatively constant in all groups, showing no substantial shift regardless of the kind of cues that the participants received. By investigating the roles of two common types of information about food and dietary change, the results contribute to discussions about how and by whom effective and efficient measures can be implemented to transform food habits. The results suggest there is limited potential for sustained and substantial behavioural changes through both social and factual information campaigns.

## Introduction

Food habits are remarkably ingrained and resistant to change. Many public and private actors use information campaigns, public guidelines, and recommendations that rely on (scientific) facts as go-to strategies aiming to change people’s eating behaviours. They are relatively inexpensive and have the advantage of being non-intrusive to individual liberty. However, the effects are in many ways disappointing. For example, while the intake of (red) meat and other animal-based products, particularly beef, has been identified and publicly broadcast as a relevant factor for both negative health and environmental impacts,^(1,2)^ global consumption is increasing.^(3)^

Given the strongly routinised and habitual nature of eating^(4)^ and its centrality in tradition, social relations, and identity,^(5)^ this is not surprising. Yet, it means that other strategies, rather than mere guidelines and recommendations, are needed if change is to be realised. One such solution could be to move from fact-based guidelines to exploiting human sociality. A broad range of research demonstrates that behavioural change, including eating patterns, is greatly affected by different forms of social influence.^(6,7)^ Social norms, social facilitation, shared identities (i.e. social homophily, in-group identification, etc.), role models and ‘central persons’ are factors that could affect human behaviour,^(8–10)^ including whether, what, when and how much individuals eat.^(11–14)^ Furthermore, several studies suggest that social information, that is, cues about what others are doing, is more successful at making people adopt or change behaviours than ‘neutral’ facts and factual information. Examples include consumer goods such as music streaming,^(15)^ energy consumption,^(16)^ towel use in hotels,^(17)^ and voter turnout.^(18)^

Most of the studies about eating behaviour are short-term interventions that do a good job at isolating different explanatory variables, yet they say little about longer-term habits. Nor do they systematically explore the potential effects of multiple co-occurring social and informational influences — which is what tends to happen in day-to-day life. In this study, we present a randomised controlled trial (RCT) in which we incorporate such co-occurring influences and explore the comparative effects of factual versus social information on eating behaviour over a period of four months. We are guided by two research questions: (1) Do social cues (social information) affect the self-reported intake of food and, if so, under what conditions? (2) Do these effects differ from the (potential) effects of factual information (e.g. dietary guidelines)?

Our pre-established main hypothesis is that social information has a larger effect on change in individual eating behaviour than factual information.^(19)^ To test this hypothesis, we study whether either of the respective kinds of information could make individuals change their self-reported intake of plant foods (increased) and animal-based foods (decreased). We are mainly inspired by the work of Damon Centola^(10,20)^ who used artificial social network experiments in online communities and demonstrated that social influences can cause *social* change, i.e., change on aggregate, not just for individuals. More precisely, he showed that repeated social reinforcement is the most effective way of driving diffusion of health behaviour in social groups and networks.

Building on this, we compare the effect of social cues with cues based on health and dietary facts. We choose to focus on plant- versus animal-based foods as an example of a dietary change that is widely discussed and emphasised as necessary to tackling both contemporary health and ecological challenges.^(3)^ With this in mind, we aim to mimic a real-world everyday life setting and contribute original insights into the ongoing discussions of how and by whom effective and efficient measures could be implemented for achieving large-scale dietary change.

## Methods

### Study design and population

The study is based on a four-month RCT using a mixed experimental design (within- and between-subjects design) in Sweden from mid-October 2022 to early February 2023. We used a mobile phone app called ‘PAN Sweden’ (henceforth referred to as ‘the app’). The app was co-developed by Metabite (previously Wellness Foundry) and Wellmo, and tailored to our needs so that it served as both an assessment tool for dietary intake and an instrument of exposure. A four-month study period was chosen based on an assessment of academic discussions about the optimal balance between studying habit change and reasonable levels of risk for dropouts and declining adherence by study participants.^(21–26)^

We tested the method and app functionalities in a pilot study with a small group of people (n=23) during two weeks in the summer of 2022. Our primary aim was to try out the registration and randomisation procedures and the app functionalities, which permitted us to test the various measurement instruments and forms of treatment. Given that this pilot study was about functionalities, we had no intention of publishing our results.

Our study population comprises adults (≥18 years old) living in Sweden, a country with a wide supply of plant-based foods, public recommendations and guidelines to consume more fruits, vegetables and whole grains while limiting the intake of (red) meat, and a political rhetoric claiming to be a country at the forefront of environmental sustainability.^(27)^ Nonetheless, meat consumption continues to be the standard and is high.^(28–31)^ The participants were recruited on different websites and physical premises (e.g. university campuses), social media channels, and through interviews with the research team in a newspaper, a podcast and on public radio. We presented the study as being about the role of information and social interaction in affecting food habits. Anyone interested could then proceed to do a screening survey. The target sample size was determined based on an extensive literature review of similar kinds of studies, suggesting a target of around 100 people *per group*.^(19)^

In the screening survey, the respondents had to confirm their age and that they were neither following a specific diet for medical reasons nor suffering from disordered eating (self-diagnosed or diagnosed by a physician). If they fulfilled the requirements, after having provided their written informed consent, they were redirected to a pre-study questionnaire that collected sociodemographic information and information about their food consumption, focusing on animal- and plant-based protein sources. Upon completion, they were redirected to a separate website that gave them contact information for receiving an anonymised login to the app. The participants did not receive any monetary compensation. However, in week 11 we announced that we would be offering a gift as token of our gratitude, and those participants who remained actively involved until the end of the study received a voucher for two meat-substitute products available at supermarkets in Sweden. The Swedish Ethical Review Authority deemed the study exempt from ethical review (dnr: 2022-02646-01).

### Exposure and assessment

The recruited participants were randomised to one of three groups using a digital die toss (www.random.org): (1) one treatment group in which the participants were exposed to factual information; (2) a second treatment group in which the participants were exposed to social information; and (3) a control group that received no information. In the app, the participants could post pictures and texts about the meals they ate and view one another’s posts within the group (but not across groups). Moreover, in all three groups network interaction was possible, meaning they could not only make posts themselves but also comment and ‘like’ each other’s activities. The structure of the design is outlined in Table 1.

**Table 1.**
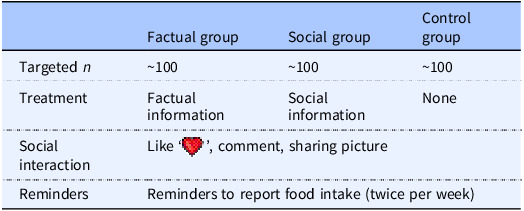
Outline of the study design

The factual information group received messages about the connection between diet and health or environmental impact based on the official Swedish dietary guidelines,^(32)^ country-specific estimates of food-related greenhouse gas emissions,^(33)^ and information about organic farming.^(34)^ The social information group received descriptive messages about what the others in the group had eaten during the past week, for example:

Over the past few weeks, your group has reported reduced consumption of dairy products. The average participant reported around seven servings in week 4, around 6 in week 5, and around 5 in week 6.

**Fig. 1.**
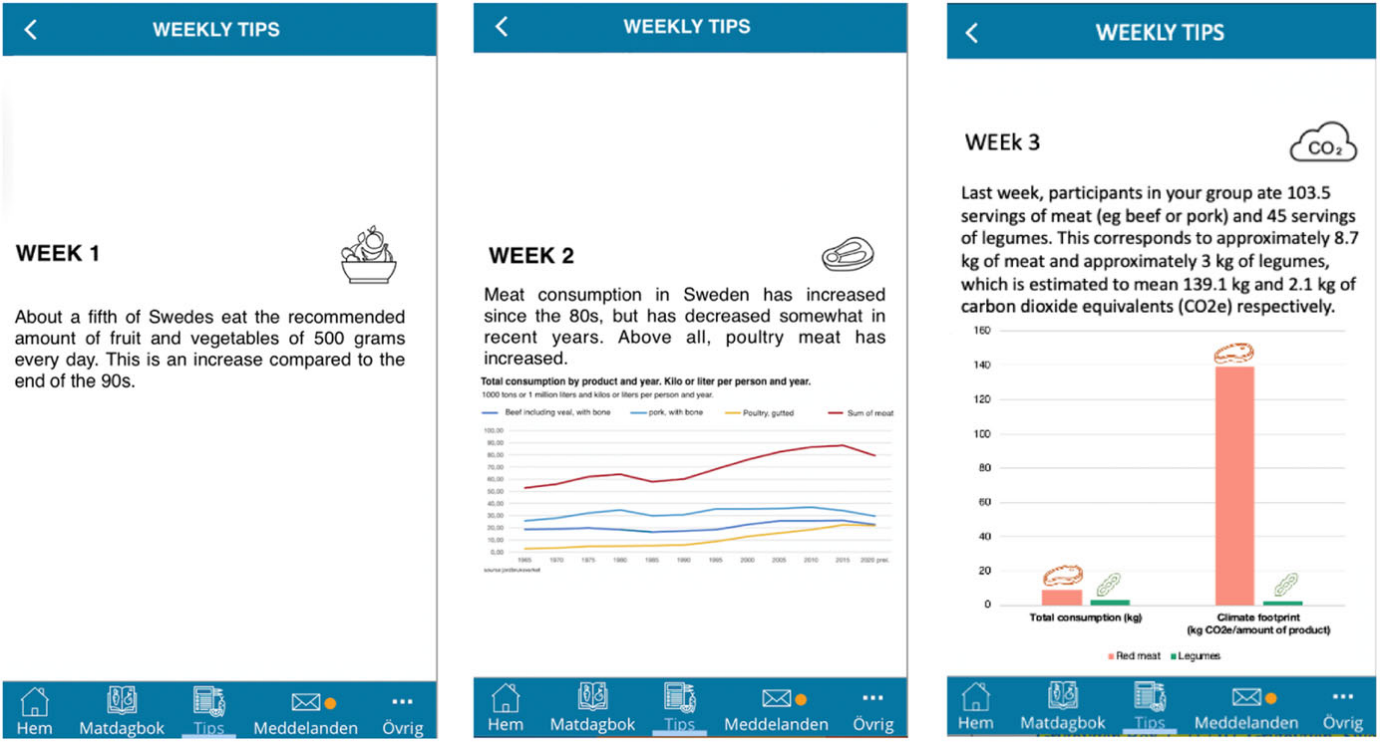
Social messages from weeks 1 (left), 2 (middle), and 3 (right) (translated from Swedish). *Source*: The sources of information for weeks 1 and 2 were the Swedish Food Agency (2012) and the Swedish Board of Agriculture (2022).

For the social group, in the first two weeks data about past consumption in the group was not available yet (week 1) or slightly skewed (week 2). Therefore, the participants received pre-established messages about food consumption in the Swedish population based on data from the Swedish Food Agency and the Swedish Board of Agriculture (see Fig. 1). From week 3 onwards, we used the true consumption data of the group (e.g. the message on the right in Fig. 1). We did not focus exclusively on decreased consumption trends, but also on both increased consumption (e.g. week 5) and neutral trends (e.g. week 8). Several messages also considered consumption patterns over multiple weeks rather than only focusing on the past week. All messages were primarily about encouraging the consumption of plant foods, but not exclusively; for example, we included information about salt intake and healthy fats (see Table S1 in the Supplementary Material).

Our intention was to mimic a real-world environment with varied information, yet plenty of (implicit) suggestions to increase plant- and decrease animal-based food intake (especially red meat). We thereby aimed to reduce the artificiality of the information flow, while also somewhat concealing the aim of the research. The data collection and subsequent analyses were not blinded to the conditions of the experiments.

The participants reported what they ate in the app by selecting portions of pre-established food categories. The food categories included in our analyses were dairy products, (red) meat, poultry, eggs, fish/shellfish, grains, legumes, fruits, starchy vegetables (e.g. potatoes), other vegetables, and nuts and seeds. For each of them, the size (i.e. gram) per portion was pre-established in the app. The participants could select as many portions per meal and food category as they had to reflect the actual amount of the food in their meal. They were asked to report a complete day’s intake at least two days per week (yet they could report as many as they wished). This was meant to balance regular reporting without overburdening them. To ensure continued participation, we sent two reminders to record food intake per week on a ‘rolling schedule’ (Tuesday/Friday, Wednesday/Saturday, etc.), so the reminders were not limited to potentially busy days. We thereby aimed to decrease the risk that the participants adjusted their food intake (e.g. eating ‘healthier’) on the days that the reminders were expected. Extra reminders were sent by SMS at mid-study time, right before New Year, and at study end (i.e. weeks 8, 11, and 16).

The study ended with a post-study questionnaire asking for the participants’ food consumption (same as the pre-study, plus questions about eating out and who usually decided what meals were eaten), as well as study involvement (e.g. perception of weekly reminders, following others in the group). This was collected for pre- and post-study comparisons, as well as to help assess interaction with other people and their posts in the app (see Table S2 in the Supplementary Material), degree of study compliance, and hidden variable biases. We also asked the participants to guess what the study was about. We did not reveal the purpose of the study in the questionnaire, but the participants had the option to contact us and find out about it after the study had ended.

### Measurements and statistical analysis

Based on the daily meal reports, we calculate our main outcome in terms of the *proportion* of plant- to animal-based foods at baseline compared to the end of the trial. For reasons of analytic transparency, the main outcome has been presented in advance,^(19)^ and is measured as the proportion of animal-based food consumption portions reported (dairy products, eggs, meat, poultry, and seafood) relative to plant food consumption portions (fruits, grains, legumes, starchy vegetables, non-starchy vegetables, and nuts and seeds) collected during the last 15 weeks of the 16-week trial. Data from week 1 is excluded due to the ongoing recruitment of participants and minor technical difficulties in the app that needed fixing.

We focus on proportions rather than absolute quantities since this outcome is less sensitive to increased/decreased amounts of food reported in total. For example, if a participant ate decreasing amounts of food in general over the study period, a decline in the total amount of meat reported could be wrongly interpreted as an exposure effect if we do not consider the general reported intakes. In addition, for both the main outcome variable and our secondary outcome (the *number* of portions reported), we use the sum of weekly food portions for each food category reported by a person, divided by the number of days recorded by the same person in each week.

The main results are initially assessed through a mixed-model repeated-measures ANOVA with unbalanced data. To explore the treatment effects on food intake, we compute the interaction effects between time (15 weeks) and treatment status (control and treatments), while the participants are applied as a random effect. Further analyses are conducted using profile analyses to determine the presence of patterns and group effects based on complete data (factual group: n = 10, social group: n = 14, control group: n = 12). Profile analysis uses techniques to identify, quantify and interpret the differences in score profiles among individuals or groups, thereby helping to examine and interpret levels or patterns of performance across multiple variables.^(35)^

We also perform explorative analyses of intakes reported in the pre- and post-questionnaires using paired sample *t*-tests for each group. The mixed-model for repeated-measures ANOVA and paired sample *t*-tests are performed using XLstat (Addinsoft, New York, USA), whereas the profile analyses are conducted in R version 4.3.1 using the profileR package (version 0.3-5). The final selection of statistical techniques was developed in consultation with a professional statistician and differs from the original plan outlined in the study protocol.^(19)^

## Results

Table 2 summarises the figures of the recruitment process: 328 received an invitation to download the app, 259 successfully registered, and 237 reported at least one meal. The distribution of dropouts at the beginning was even across the three groups, suggesting that the randomisation worked well. While lower than the targeted number of participants per group (i.e. 100), we reached about 80 individuals per group who reported two to three meals on two or more days per week. 16 participants actively dropped out, i.e., stating they wanted to withdraw from the study.

**Table 2.** Recruitment and participation in the study groups

Group	Invited	Accessed	Reported at least one meal
Factual	110	88	79
Social	110	91	83
Control	108	80	75
Total	328	259	237

*Note*: The ‘Invited’ column refers to participants who received log-in details for the app, while the ‘Accessed’ column refers to participants who successfully downloaded and logged in to the app.

The mean age of the participants who reported at least one meal was 42 (range: 20–74 years), and the average monthly income report is in line with the Swedish average (Table 3; see also Table S3 in the Supplementary Material). The sample is skewed in terms of gender and level of education with 76 per cent being female and 78 per cent reporting a college/university degree or higher. Nevertheless, the consumption of meat, poultry and other animal-based protein sources was surprisingly high.

**Table 3.** Characteristics of participants who reported a meal at least once

	Total	Factual	Social	Control
N (%)	237 (100%)	79 (33%)	83 (35%)	75 (32%)
Gender				
Female	182 (76%)	55 (70%)	66 (80%)	60 (80%)
Male	54 (24%)	24 (30%)	17 (20%)	15 (20%)
Age: mean (SD)	42 (±11)	41 (±12)	41 (±11)	44 (±10)
Education				
Upper secondary school education	37 (16%)	15 (19%)	8 (10%)	14 (19%)
Post-upper secondary school education	15 (6%)	7 (9%)	6 (7%)	2 (3%)
College/university education	174 (73%)	54 (68%)	63 (76%)	57 (76%)
Postgraduate education	11 (5%)	3 (4%)	6 (7%)	2 (3%)
Monthly income				
Less than SEK 15,000	22 (9%)	10 (13%)	4 (5%)	8 (11%)
SEK 15,000–34,999	69 (29%)	22 (28%)	24 (29%)	23 (31%)
SEK 35,000–49,999	110 (46%)	39 (49%)	39 (47%)	32 (43%)
SEK 50,000 and more	33 (14%)	8 (10%)	14 (17%)	11 (15%)
Prefer not to say	3 (1%)	0 (0%)	2 (2%)	1 (1%)
Dietary orientation				
Omnivore	172 (73%)	53 (67%)	68 (82%)	51 (68%)
Flexitarian	33 (14%)	13 (16%)	10 (12%)	10 (13%)
Pescatarian	9 (4%)	4 (5%)	2 (2%)	3 (4%)
Vegetarian	12 (5%)	4 (5%)	2 (2%)	6 (8%)
Vegan	9 (4%)	5 (6%)	1 (1%)	3 (4%)
Other	2 (1%)	0 (0%)	0 (0%)	2 (3%)
Consumption frequency^a^				
Animal-based protein sources				
* Never*	24 (10%)	11 (14%)	4 (5%)	9 (12%)
* Less than once a week*	10 (4%)	2 (3%)	4 (5%)	4 (5%)
* Once or twice a week*	31 (13%)	11 (14%)	13 (16%)	7 (9%)
* Three or four times a week*	50 (21%)	13 (16%)	20 (24%)	17 (23%)
* Five or more times a week*	122 (51%)	42 (53%)	42 (51%)	38 (51%)
Plant-based protein sources				
* Never*	24 (10%)	9 (11%)	10 (12%)	5 (7%)
* Less than once a week*	78 (33%)	19 (24%)	31 (37%)	28 (37%)
* Once or twice a week*	69 (29%)	23 (29%)	26 (31%)	20 (27%)
* Three or four times a week*	42 (18%)	18 (23%)	10 (12%)	14 (19%)
* Five or more times a week*	24 (10%)	10 (13%)	6 (7%)	8 (11%)
Meat alternatives				
* Never*	53 (22%)	20 (25%)	16 (19%)	17 (23%)
* Less than once a week*	84 (35%)	21 (27%)	33 (40%)	30 (40%)
* Once or twice a week*	55 (23%)	18 (23%)	24 (29%)	13 (17%)
* Three or four times a week*	30 (13%)	14 (18%)	6 (7%)	10 (13%)
* Five or more times a week*	15 (6%)	6 (8%)	4 (5%)	5 (7%)

*Note*: All data are from the pre-questionnaire.

a Animal-based protein sources include beef, pork, lamb, poultry, seafood; plant-based protein sources include beans, peas, quinoa, etc; meat alternatives include vegetarian meat alternatives such as mycoprotein products and soya mince.

Seventy-three per cent of the participants categorised themselves as omnivores, 14 per cent as flexitarian, and only 5 per cent claimed to follow a vegetarian diet. The rate of a vegetarian diet is slightly higher than suggested for the Swedish population.^(28)^ Vegan and pescatarian diets are followed by 4 per cent each, also higher than national estimates. At the same time, 51 per cent reported that they consumed animal-based protein sources five or more times per week, 21 per cent 3 or 4 times per week, and 13 per cent once or twice per week. More than half of the participants reported substituting meat with plant protein sources such as legumes and pulses on a weekly basis or more often, while 10 per cent never did this. In contrast, 22 per cent reported that they never used meat substitutes, while around 40 per cent reported using them on a weekly basis (most of them — 23 per cent — once or twice a week).

**Fig. 2.**
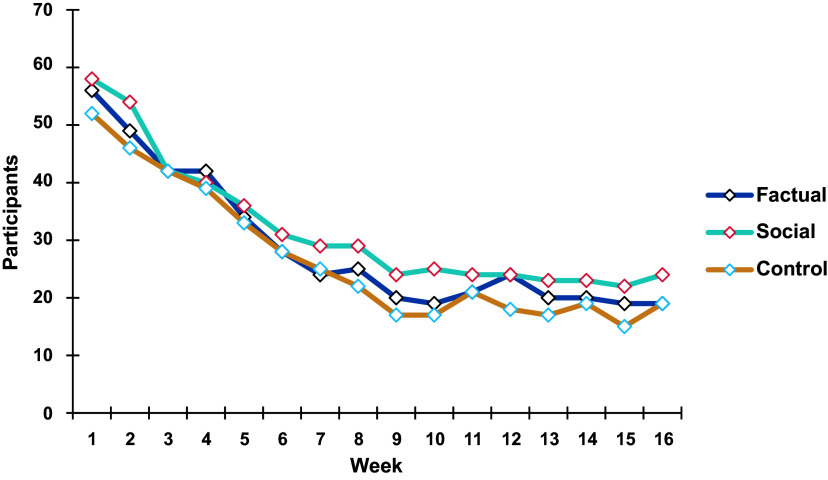
The number of participants who reported meals per week over time.

During the study, an average of 310 meals were logged per week. This includes a total of 6,039 meals reported in treatment group 1 (factual group), 4,487 meals in group 2 (social group), and 4,353 in the control group over 16 weeks. In the first weeks, reporting and posting of meals was strong across all groups. As the study progressed, activity decreased substantially over time and ensued slightly differently across the groups, as can be seen in Fig. 2 (see also Table S4 in Supplementary Material). At the study end, around 21 participants continued to regularly report their meals in each group.

### Main outcomes

**Fig. 3.**
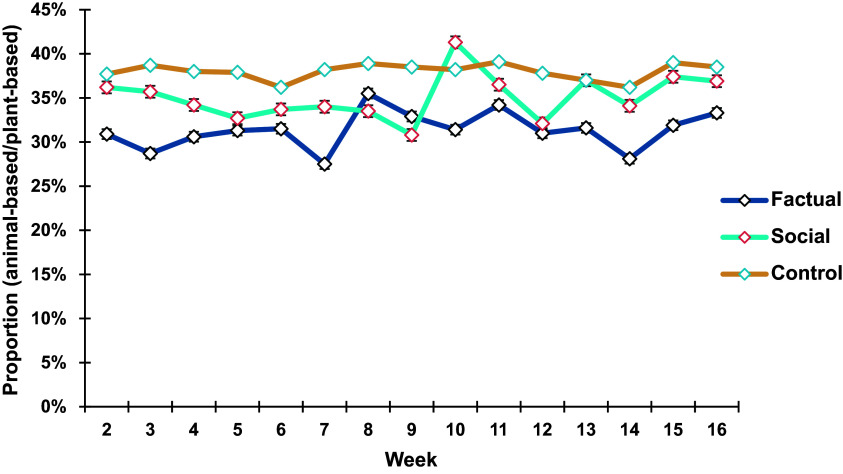
Proportion of animal-based foods (vs. plant-based foods) consumption over time (± standard error, SE).

The proportions of food consumption (in portions) of animal-based foods compared to plant-based foods over time are shown in Fig. 3. All three groups experienced some fluctuations over time, but there is no statistically significant effect at the group level (F(2, 159.4) = 2.694, P = 0.071) or at the week level (F(14, 1044.3) = 1.190, P = 0.277). No interaction effect between group and week can be observed (F(28, 1044.1) = 0.966, P = 0.516). Although the P-value does not reach conventional levels of significance, the F-statistic suggests some variability between groups. Therefore, an additional profile analysis with complete data is performed. But the results do not change (see Table S5 in the Supplementary Material).

### Secondary and explorative outcomes

Despite the absence of significant effects on the proportions of food consumption, further analyses are performed on the absolute intake of animal-based and plant-based food over the study period. The mixed model for repeated measures ANOVA results reveal that, at the *group* level, there is no statistically significant difference in the consumption of either animal-based food consumption (F(2, 168.5) = 2.192, P = 0.115) or plant-based food consumption (F(2, 160.8) = 2.082, P = 0.128). However, statistically significant *week* effects can be observed for both animal-based (F(14, 1058.6)=2.029, P = 0.013) and plant-based foods (F(14, 1053.8) = 2.227, P = 0.006), indicating a significant difference across the weeks. No significant interactions in food consumption are detectable between group and week for both animal-based (F(28, 1058.5) = 0.840, P = 0.706) and plant-based foods (F(28, 1053.5) = 0.892, P = 0.628).

**Fig 4.**
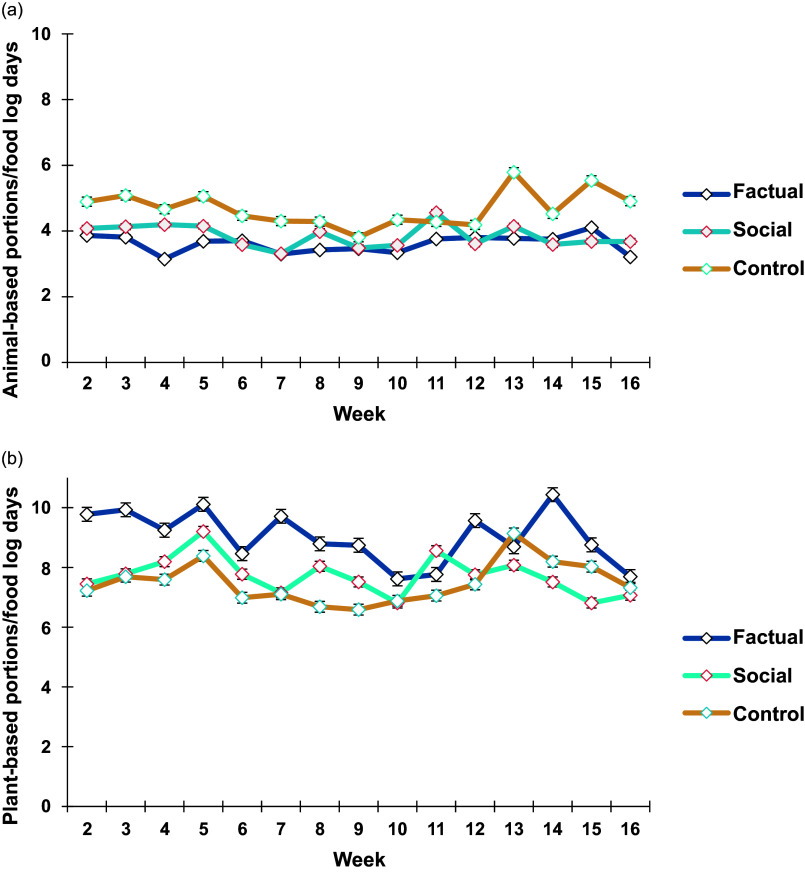
Average food consumption portion divided by food log days per week (± standard error, SE). Graphs are shown for animal-based foods (a) and plant-based foods (b).

Further analyses with profiles reveal similar patterns of animal-based food consumption for all three groups. However, average animal-based food consumption (i.e. the portions) differ across weeks (F(14, 20) = 2.814, P = 0.017; see Table S6). This is graphically depicted in Fig. 4a and b: All three groups experienced fluctuations in their consumption of animal-based food (considering food log frequencies), with some peaking in the middle of the study period (around Christmas). The control group reported consistently higher amounts compared to the factual and social groups, ending at the level where it started (approx. 4.9 portions) after a stronger peak around the Christmas holidays. The two treatment groups, in turn, show a relatively flat pattern, though with a slight decline compared to the start of the study (social group: approx. 4.1 portions down to 3.7; factual group: approx. 3.9 portions down to 3.2).

The consumption of plant-based food shows more volatility in all groups, including several peaks and dips. A profile analysis of plant-based food consumption validates this impression, showing comparable patterns across the three groups and an overall average that remained relatively consistent across weeks (F(14, 20) = 1.303, P = 0.287). However, the average plant-based food consumption differs at the group level (F(2,33) = 4.185, P = 0.024; see Supplementary Material, Table S7). Specifically, it was higher in the factual group compared to the other two groups and slightly decreased from the beginning to end of the study period, thus towards the end approaching the level of the other two groups. In summary, we find weekly fluctuation but no substantial longer-term changes within or across groups.

Lastly, we explore the frequencies of meat and plant-based food consumption in the pre- and post-questionnaires (not pre-established in the study protocol). The results are shown in Table 4. In the social group, the frequency of meat consumption decreased from ‘three to four times a week’ closer to ‘once or twice a week’ (4.2 and 3.8; P = 0.042). No other statistically significant changes occurred in the three groups, not even an increase in plant foods for the same group (social group) that reported lower follow-up numbers for meat, accounting for the decline in reporting over the weeks.

**Table 4.** Frequency of meat consumption and plant-based food consumption changes between pre- and post-questionnaires

	Factual			Social			Control		
	19			22			18		
N	Pre	Post	P-value	Pre	Post	P-value	Pre	Post	P-value
Meat consumption (frequency)	3.4 (±1.5)	3.5 (±1.6)	0.772	4.2 (±1.2)	3.8 (±1.2)	**0.042**	4.4 (±1.1)	4.3 (±1.1)	0.772
Plant-based food consumption (frequency)	3.3 (±1.2)	3.3 (±1.5)	0.858	3.0 (±1.2)	3.0 (±1.0)	1.000	2.8 (±1.0)	2.9 (±1.2)	0.790

*Note*: the P-value was calculated using a paired *t*-test. The figures represent means (SD). The answer scale in the questionnaire was: 1 = ‘Never’, 2 = ‘Less than once a week’, 3 = ‘Once or twice a week’, 4 = ‘Three or four times a week”, and 5 = ‘Five or more times a week’.

## Discussion

To restore and sustain human and planetary health, a fundamental social change in diets is needed.^(2,36,37)^ Public official^(38)^ and unofficial dietary guidelines^(39)^ promote dietary transformation towards an increased proportion of (healthy) plant foods. The results of our four-month RCT with adults in Sweden suggest that both our treatment approaches — providing factual information or social cues in the form of the consumption of other group members — are unsuccessful in achieving this. There were indeed weekly fluctuations, indicating that some information was perhaps more effective than other. Yet, the first analyses did not reveal any statistically significant effects, and it is difficult to establish in this study whether observed fluctuations were attributable to a certain information message because participants could view previous messages accumulated as weekly tips in their app (thus, the effectiveness of a specific message could vary over time). Also, we can see a slight decrease in the consumption of animal-based food in the group that received social information, as found in the exploratory analysis. However, in general, the overarching picture is that the participants did not change their eating habits despite 16 weeks of regular external influence through the app.

Our results could be attributable to methodological problems such as the declining level of participation, or perhaps even due to an inadequate sample size from the outset. It could also be that messaging information on a weekly basis was not intense enough, that is, too infrequent or too mild, compared to, for example, giving the participants more alarming messages on a daily basis. However, we aimed to embed the factual messages into a similar context to what (Swedish) individuals are actually exposed to, and we wanted the social messages to be true. Another explanation could be the form of social influence. As Centola^(10)^ has convincingly demonstrated, the complex contagion of behaviour is most effectively diffused through strong ties, but the interaction and influence of this study could only be considered weak. Also, the messages may have been too general, activating social norms but lacking the personalised impact necessary for (stronger) social effects. Incorporating injunctive norms, which signal approval of desirable behaviours, might have further strengthened the intervention by reinforcing positive actions among those participants who were already consuming less animal-based foods.^(40)^

The fact that the study collided with the holiday period around Christmas and New Year must also be acknowledged, as this time of year tends to centre on meat-intensive foods (popular Swedish holiday dishes include ham, meatballs, and fillet of beef). The data do suggest a minor holiday effect, partly in what was reported to be eaten, but even more by a lower level of activity. Nonetheless, we managed to re-activate the participants by sending them reminders, and the reported eating patterns once again regressed towards normality. This underlines the nature of eating as a strongly habituated and routinised practice, and it makes the results plausible.

With this in mind, the study makes a theoretical and methodological contribution. Great hopes of achieving large-scale dietary change and other forms of healthy or environmentally-friendly behaviours are often put in behaviour-focused ‘nudges’, which seem reasonably effective in the short term,^(41–45)^ when embedding descriptive social norms in informational campaigns^(16)^ or exposing people to social reinforcement diffusion through social networks.^(9,10)^ Our results suggest that hope or belief in the power of social and informational mechanisms to induce lasting change should be toned down, at least as far as such ‘stubbornly’ persistent behaviours as everyday eating habits are concerned. As comparable developments in the control and treatment groups indicate, such influences can indeed make people choose differently at single moments, something that is supported by a wide array of (experimental) research.^(11–14,46–48)^ However, the deeply habitual and routinised dietary patterns in everyday life outside the controlled laboratory setting appear to be substantially harder to influence by regular exposure to both factual and social information.

Having said that, our study reflects some common problems of longitudinal RCTs, facing decreasing activity among participants, and sample sizes that are both smaller and more demographically skewed than what would be optimal. However, the randomisation process seems to have worked very well, the hypothesis and the main outcome variables are clear, as are the treatments. The methodological approach and the behavioural mechanisms it can study are also not limited to questions about plant/animal foods or diets. The study design appears to be well replicable in our own validation studies and in studies of realms of behaviour, such as consumer goods purchasing, transportation, or exercising. Equally, our study may serve to validate previous studies with similar comparisons.^(16–18,49)^ Since this study is limited by the portion options available in the app — sufficient to test the behavioural hypothesis, but not to evaluate the nutritional or environmental metrics in detail — studies using more sophisticated measurement options can hopefully build on ours. It could also be extended socially, such as applying it to already existing networks with no anonymity, or groups that are anonymous but in which the networking opportunities are developed in ways that allow for more complex social interaction, thereby exploiting the power of strong ties.

Lastly, a unique methodological contribution is the use of the app as an instrument of both assessment and exposure. This type of technology has become very influential as a means of collecting data or offering behavioural ‘coaching’, for example, on physical activity and diets,^(50)^ but it is under-appreciated for its potential to also act as an experimental instrument for longitudinal behavioural treatment. This is what we have done and while greatly inspired by existing studies, we present a method that stands out in the literature. We have also been transparent about our hypotheses and planned outcomes of interest from our study protocol^(19)^ in order to counter the serious issues of p-hacking and file drawer problems that are plaguing the sciences.^(51)^

## Conclusion

In conclusion, in our four-month RCT that tested the effects of factual information versus social cues on self-reported dietary behaviour we observe neither between- nor within-group differences over time. The pre- and post-study comparison hints at a possible effect of social information, but the explorative nature of this analysis warrants particular care in its interpretation. We believe that our results confirm the stubbornly persistent nature of eating behaviour as a strongly habituated, routinised, and symbolically meaningful practice. This provides insights into ongoing research that tries to discern ways of advancing social change to tackle current health and ecological crises. It suggests toning down hopes in the potential of ‘the informed social crowd’ to bring about substantial change. If this is true, socio-political energy will likely be more effectively placed in less ‘laissez-faire’ approaches. Furthermore, with our method of using a mobile phone app as an instrument of both behavioural assessment and exposure, we think we provide an important contribution to the broader field of health behaviour. We hope to inspire more social scientists to use the potential of RCTs to dive deeper into the causal mechanisms of individuals and societal behaviour change.

## Supporting information

Zorell et al. supplementary material Zorell et al. supplementary material

## Supplementary material

The supplementary material for this article can be found at https://doi.org/10.1017/jns.2024.82
