# Supplementary material for: Studying factual versus social cues as triggers of change in food behaviour
Source: J Nutr Sci. 2024 Dec 3;13:e88. doi: 10.1017/jns.2024.82 (PMC11658939; doi:10.1017/jns.2024.82)
Supplement: Zorell et al. supplementary material [file S204867902400082Xsup001.docx]

**Table S1: Contents of the messages sent to the treatment groups**

| Week | Theme | Factual message | Social message |
| --- | --- | --- | --- |
| 1 | Health | Fruit and vegetables  (Official dietary advice) | Swedes’ fruit and vegetable   consumption |
| 2 | Environment | CO_2_e per protein sources | Swedes’ meat consumption |
| 3 | Health | Type of cooking fat  (Official dietary advice) | Consumption of meat and legumes  (Real time-based update) |
| 4 | Health | Amount of meat  (Official dietary advice) | Consumption of fruits and vegetables  (Real time-based update) |
| 5 | Environment | Recommended type of meat  (Official dietary advice) | Consumption of meat and legumes  (Real time-based update) |
| 6 | Health | Nuts and seeds  (Official dietary advice) | Animal- and plant-based food consumption and land use  (Real time-based update) |
| 7 | Environment | CO_2_e at different food proportions | Consumption of dairy products  (Real time-based update) |
| 8 | Environment | Organic farming | Consumption of legumes and starchy vegetables  (Real time-based update) |
| 9 | Health | Whole grains  (Official dietary advice) | Consumption of fats and grains  (Real time-based update) |
| 10 | Environment | Less meat  (Official dietary advice) | Average proportion of each food group on the plate  (Real time-based update) |
| 11 | Environment | Type of cooking fat  (Official dietary advice) | Consumption of caffeine and sugar  (Real time-based update) |
| 12 | Environment | CO_2_e for ready meals | Meat and legume consumption and corresponding CO_2_e  (Real time-based update) |
| 13 | Health | Whole grains  (Official dietary advice) | Food consumption over the holidays  (Real time-based update) |
| 14 | Health | Amount of meat  (Official dietary advice) | Consumption of meat, legumes, and vegetables  (Real time-based update) |
| 15 | Environment | CO_2_e for protein sources | Meat and legume consumption and corresponding CO_2_e  (Real time-based update) |
| 16 | Health | Amount of salt  (Official dietary advice) | Consumption of meat, legumes, eggs, dairy products, and fats  (Real time-based update) |

**Table S2: Extent to which participants followed others’ posts**

|  | Factual (n=19) | Social (n=24) | Control (n=18) | *p*-value |
| --- | --- | --- | --- | --- |
| Very incorrect | 2 (11%) | 7 (32%) | 9 (50%) | 0.129 |
| Fairly incorrect | 7 (36%) | 9 (41%) | 5 (28%) |  |
| Fairly correct | 8 (42%) | 3 (14%) | 3 (17%) |  |
| Very correct | 2 (11%) | 3 (14%) | 1 (5%) |  |

Note: The responses come from the post-questionnaire. Participants where asked, to what extent they think that the following statement applies to them: “I followed what other participants in my group reported”.

**Table S3: Characteristics of individuals who registered for the study**

|  | **Total** | **Factual** | **Social** | **Control** |
| --- | --- | --- | --- | --- |
| **N** (%) | 328 (100%) | 110 (33.5%) | 110 (33.5%) | 108 (33%) |
| **Gender** |  |  |  |  |
| Female | 258 (79%) | 84 (76%) | 86 (78%) | 88 (81%) |
| Male | 70 (21%) | 26 (24%) | 24 (22%) | 20 (19%) |
| **Age: mean (SD)** | 44 (±13) | 43 (±13) | 43 (±13) | 47 (±12) |
| **Education** |  |  |  |  |
| Primary school education | 1 (0.3%) | 0 (0%) | 0 (0%) | 1 (1%) |
| Upper secondary school education | 53 (16%) | 22 (20%) | 13 (12%) | 18 (17%) |
| Post-upper secondary education | 26 (8%) | 8 (7%) | 10 (9%) | 8 (7%) |
| College/university education | 230 (70%) | 75 (68%) | 79 (72%) | 76 (70%) |
| Postgraduate education | 18 (5%) | 5 (5%) | 8 (7%) | 5 (5%) |
| **Monthly income** |  |  |  |  |
| Less than SEK 14,999 | 26 (8%) | 12 (11%) | 5 (5%) | 9 (8%) |
| SEK 15,000 – 34,999 | 110 (34%) | 36 (33%) | 37 (34%) | 37 (34%) |
| SEK 35,000 – 49,999 | 137 (42%) | 51 (46%) | 45 (41%) | 41 (38%) |
| Over SEK 50,000 | 48 (15%) | 11 (10%) | 19 (17%) | 18 (17%) |
| Prefer not to say | 7 (2%) | 0 (0%) | 4 (4%) | 3 (3%) |
| **Dietary orientation** |  |  |  |  |
| Omnivore | 242 (74%) | 76 (69%) | 87 (79%) | 79 (73%) |
| Flexitarian | 45 (14%) | 17 (15%) | 14 (13%) | 14 (13%) |
| Pescatarian | 11 (3%) | 6 (5%) | 2 (2%) | 3 (3%) |
| Vegetarian | 14 (4%) | 5 (5%) | 3 (3%) | 6 (6%) |
| Vegan | 13 (4%) | 6 (5%) | 4 (4%) | 3 (3%) |
| Other | 3 (1%) | 0 (0%) | 0 (0%) | 3 (3%) |
| **Consumption frequency*** |  |  |  |  |
| Animal-based protein sources |  |  |  |  |
| *Never* | 31 (9%) | 14 (13%) | 8 (7%) | 9 (8%) |
| *Less than once a week* | 15 (5%) | 2 (2%) | 7 (6%) | 6 (6%) |
| *Once or twice a week* | 46 (14%) | 14 (13%) | 17 (15%) | 15 (14%) |
| *Three or four times a week* | 73 (22%) | 23 (21%) | 23 (21%) | 27 (25%) |
| *Five or more times a week* | 163 (50%) | 57 (52%) | 55 (50%) | 51 (47%) |
| Plant-based protein sources |  |  |  |  |
| *Never* | 30 (9%) | 12 (11%) | 12 (11%) | 6 (6%) |
| *Less than once a week* | 112 (34%) | 29 (26%) | 43 (39%) | 40 (37%) |
| *Once or twice a week* | 102 (31%) | 33 (30%) | 34 (31%) | 35 (32%) |
| *Three or four times a week* | 55 (17%) | 25 (23%) | 13 (12%) | 17 (16%) |
| *Five or more times a week* | 29 (9%) | 11 (10%) | 8 (7%) | 10 (9%) |
| Meat alternatives |  |  |  |  |
| *Never* | 78 (24%) | 30 (27%) | 20 (18%) | 28 (26%) |
| *Less than once a week* | 121 (37%) | 33 (30%) | 46 (42%) | 42 (39%) |
| *Once or twice a week* | 78 (24%) | 24 (22%) | 32 (29%) | 22 (20%) |
| *Three or four times a week* | 34 (10%) | 17 (15%) | 7 (6%) | 10 (9%) |
| *Five or more times a week* | 17 (5%) | 6 (5%) | 5 (5%) | 6 (6%) |

*Animal-based protein sources include beef, pork, lamb, poultry, seafood; plant-based protein sources include beans, peas, quinoa, etc; meat alternatives include vegetarian meat alternatives such as mycoprotein products and soy mince.

Note: All data are from the pre-questionnaire.

**Table S4: The number of participants who reported meals per week over time**

| **Week** | **Factual** | **Social** | **Control** |
| --- | --- | --- | --- |
| 1 | 56 | 58 | 52 |
| 2 | 49 | 54 | 46 |
| 3 | 42 | 42 | 42 |
| 4 | 42 | 40 | 39 |
| 5 | 34 | 36 | 33 |
| 6 | 28 | 31 | 28 |
| 7 | 24 | 29 | 25 |
| 8 | 25 | 29 | 22 |
| 9 | 20 | 24 | 17 |
| 10 | 19 | 25 | 17 |
| 11 | 21 | 24 | 21 |
| 12 | 24 | 24 | 18 |
| 13 | 20 | 23 | 17 |
| 14 | 20 | 23 | 19 |
| 15 | 19 | 22 | 15 |
| 16 | 19 | 24 | 19 |

**Table S5: Results of profile analysis on the proportion of animal-based and plant-based food consumption over time**

| **Test for parallelism** |  |  |  |  |  |
| --- | --- | --- | --- | --- | --- |
| Multivariate tests | Statistic | Approx. F | num. df | den. df | *p* |
| Wilks | 0.404 | 0.820 | 28 | 40 | 0.706 |
| Pillai | 0.727 | 0.857 | 28 | 42 | 0.662 |
| Hotelling-Lawley | 1.152 | 0.782 | 28 | 38 | 0.748 |
| Roy | 0.664 | 0.996 | 14 | 21 | 0.490 |
|  |  |  |  |  |  |
| **Test for equal levels** |  |  |  |  |  |
| Univariate test | Df | Sum Sq | Mean Sq | F value | *p* |
| Group | 2 | 0.086 | 0.043 | 2.823 | 0.074 |
| Residuals | 33 | 0.502 | 0.015 |  |  |
|  |  |  |  |  |  |
| **Test for flatness** |  |  |  |  |  |
|  | F | df1 | df2 |  | *p* |
|  | 0.337 | 14 | 20 |  | 0.979 |

**Table S6: Results of profile analysis on animal-based food consumption over time**

| **Test for parallelism** |  |  |  |  |  |
| --- | --- | --- | --- | --- | --- |
| Multivariate tests | Statistic | Approx. F | num. df | den. df | *p* |
| Wilks | 0.355 | 0.969 | 28 | 40 | 0.527 |
| Pillai | 0.778 | 0.954 | 28 | 42 | 0.544 |
| Hotelling-Lawley | 1.443 | 0.979 | 28 | 38 | 0.516 |
| Roy | 1.105 | 1.657 | 14 | 21 | 0.143 |
|  |  |  |  |  |  |
| **Test for equal levels** |  |  |  |  |  |
| Univariate test | Df | Sum Sq | Mean Sq | F value | *p* |
| Group | 2 | 25.2 | 12.58 | 1.125 | 0.337 |
| Residuals | 33 | 368.9 | 11.18 |  |  |
|  |  |  |  |  |  |
| **Test for flatness** |  |  |  |  |  |
|  | F | df1 | df2 |  | *p* |
|  | 2.814 | 14 | 20 |  | **0.017** |

**Table S7: Results of profile analysis on plant-based food consumption over time**

| **Test for parallelism** |  |  |  |  |  |
| --- | --- | --- | --- | --- | --- |
| Multivariate tests | Statistic | Approx. F | num. df | den. df | *p* |
| Wilks | 0.329 | 1.062 | 28 | 40 | 0.424 |
| Pillai | 0.839 | 1.084 | 28 | 42 | 0.399 |
| Hotelling-Lawley | 1.527 | 1.036 | 28 | 38 | 0.453 |
| Roy | 1.032 | 1.549 | 14 | 21 | 0.177 |
|  |  |  |  |  |  |
| **Test for equal levels** |  |  |  |  |  |
| Univariate test | Df | Sum Sq | Mean Sq | F value | *p* |
| Group | 2 | 61.44 | 30.722 | 4.185 | **0.024** |
| Residuals | 33 | 242.28 | 7.342 |  |  |
|  |  |  |  |  |  |
| **Test for flatness** |  |  |  |  |  |
|  | F | df1 | df2 |  | *p* |
|  | 1.303 | 14 | 20 |  | 0.287 |
